# Assessment of Prevalence, Awareness, and Characteristics of Isolated Systolic Hypertension Among Younger and Middle-Aged Adults in China

**DOI:** 10.1001/jamanetworkopen.2020.9743

**Published:** 2020-12-08

**Authors:** Shiwani Mahajan, Fang Feng, Shuang Hu, Yuan Lu, Aakriti Gupta, Karthik Murugiah, Yan Gao, Jiapeng Lu, Jiamin Liu, Xin Zheng, Erica S. Spatz, Haibo Zhang, Harlan M. Krumholz, Jing Li

**Affiliations:** 1 The Center for Outcomes Research and Evaluation, Yale-New Haven Hospital, New Haven, Connecticut; 2 Yale School of Medicine, Section of Cardiovascular Medicine, Department of Internal Medicine, New Haven, Connecticut; 3 National Clinical Research Center for Cardiovascular Diseases, NHC Key Laboratory of Clinical Research for Cardiovascular Medications, State Key Laboratory of Cardiovascular Disease, Fuwai Hospital, Chinese Academy of Medical Sciences and Peking Union Medical College, National Center for Cardiovascular Diseases

## Abstract

**Importance:**

Isolated systolic hypertension (ISH) is increasing in prevalence among young and middle-aged adults. However, most studies of ISH are limited to older individuals, and a substantial knowledge gap exists regarding younger adults with ISH.

**Objective:**

To assess the prevalence, awareness, and characteristics of ISH among younger and middle-aged adults in China.

**Design, Setting, and Participants:**

This cross-sectional study was performed as part of the China Patient-Centered Evaluative Assessment of Cardiac Events Million Persons Project, which enrolled 3.1 million community residents aged 35 to 75 years from all of the 31 provinces in China between December 15, 2014, and May 15, 2019. The present analysis included only participants younger than 50 years. Data were analyzed from May to November 2019.

**Main Outcomes and Measures:**

Prevalence and awareness of ISH (defined as systolic blood pressure of 140 mm Hg or higher and diastolic blood pressure of less than 90 mm Hg) and individual characteristics of participants with ISH.

**Results:**

Among 898 929 participants aged 35 to 49 years, the mean (SD) age was 43.8 (3.9) years; 548 657 participants (61.0%) were women, and 235 138 participants (26.2%) had hypertension. Of those with hypertension, 62 819 participants (26.7%; 95% CI, 26.5%-26.9%) had ISH (mean [SD] age, 45.0 [3.5] years; 41 417 women [65.9%]), and 54 463 of those with ISH (86.7%; 95% CI, 86.4%-87.0%) had not received treatment. The prevalence of ISH was higher among individuals who were older, were female, were farmers, resided in the eastern region of China, and had an educational level of primary school or lower. Women and older individuals were more likely to have ISH than to be normotensive or to have other hypertension subtypes. Participants who were obese, currently used alcohol, had diabetes, and experienced previous cardiovascular events were more likely to have other types of hypertension and less likely to have normotension than to have ISH. Among the 54 463 participants with ISH who had not received treatment, only 3682 individuals (6.8%; 95% CI, 6.6%-7.0%) were aware of having hypertension, and awareness rates remained low even among those with systolic blood pressure of 160 mm Hg or higher (7135 individuals [13.1%; 95% CI, 12.4%-13.9%]).

**Conclusions and Relevance:**

In this study, ISH was identified in 1 of 4 young and middle-aged adults with hypertension in China, most of whom remained unaware of having hypertension. These results highlight the increasing need for better guidance regarding the management of ISH in this population.

## Key Points

**Question:**

What are the characteristics of young and middle-aged adults with isolated systolic hypertension in China, and what is the prevalence and awareness of isolated systolic hypertension among this population?

**Findings:**

In this cross-sectional study of young and middle-aged adults in China, isolated systolic hypertension was identified in 27% of participants with hypertension, 87% of whom had not received treatment; less than 7% of individuals with untreated isolated systolic hypertension were aware of having hypertension. Among individuals with isolated systolic hypertension, 16% had systolic blood pressure of 160 mm Hg or higher, but awareness rates remained low even in this group.

**Meaning:**

In this study, a substantial proportion of young and middle-aged adults with hypertension had isolated systolic hypertension; there is an opportunity to improve awareness of isolated systolic hypertension among this population.

## Teaser

This cross-sectional study includes younger and middle-aged adult participants from the China Patient-Centered Evaluative Assessment of Cardiac Events Million Persons Project to assess the prevalence, awareness, and individual characteristics of isolated systolic hypertension (ISH) among this population.

## Introduction

Isolated systolic hypertension (ISH), a subtype of hypertension, is experienced by more than 40% of adults with untreated hypertension.^1,2,3^ Among those who do receive treatment, control of systolic blood pressure (SBP) is particularly challenging compared with control of diastolic blood pressure (DBP), making ISH the most common subtype of hypertension among patients with uncontrolled hypertension.^4,5,6,7^ Isolated systolic hypertension is a well-studied condition that has received attention across various hypertension guidelines. However, ISH is disproportionately found in older adults, and many of the disease management recommendations are based on studies and data of older individuals.^1,8,9,10,11,12,13^

Young and middle-aged adults are experiencing an increasing prevalence of ISH,^14,15,16^ which can increase their risk of heart disease and stroke.^17^ Younger individuals with ISH may have distinct characteristics that require exploration given that the pathophysiologic characteristics of ISH may differ from those of older individuals (eg, aortic stiffness in older adults and increased cardiac output or stroke volume in younger adults), which can have implications for disease management.^18^ However, most previous studies of ISH have focused on older individuals (>50 years),^1,19^ and the few studies of younger individuals with ISH in China have only provided information regarding the overall prevalence of ISH in this population.^16^ Thus we lack a comprehensive understanding of the prevalence, awareness, and characteristics of young and middle-aged individuals with ISH in the Chinese population and how these factors may vary across diverse subgroups of the population.

The China Patient-Centered Evaluative Assessment of Cardiac Events Million Persons Project (China PEACE MPP), a large-scale population-based screening project, provided a suitable platform to examine ISH among young and middle-aged adults given the project’s large data set (N = 3 094 655) and recruitment of participants at the community level. We performed a cross-sectional study of young and middle-aged participants from the China-PEACE MPP to describe the prevalence, awareness, and individual characteristics of ISH among this population.

## Methods

### Study Design and Population

Details of the design of the China PEACE MPP have been described previously.^20^ In brief, between December 15, 2014, and May 15, 2019, 244 sites (146 rural counties and 98 urban districts) were selected by a convenience sampling strategy from county-level geographic regions in 31 provinces of mainland China. Participants were enrolled in the China PEACE MPP if they were aged 35 to 75 years and had a Hukou (an official record that identifies area residents) for a region selected for the study. Participants were recruited through publicity campaigns in the media and by mail. The study was approved by the central ethics committee of the China National Center for Cardiovascular Disease and the institutional review board of Yale University. All enrolled participants provided written informed consent. This study was reported in accordance with the Strengthening the Reporting of Observational Studies in Epidemiology (STROBE) reporting guideline for cross-sectional studies.^21^

Of the 3 094 655 participants enrolled in the China PEACE MPP during the study period, 899 128 young and middle-aged adults (29.1%) between ages 35 and 49 years were selected for the present cross-sectional study. We excluded 129 participants who were missing data for age and 70 participants who were missing data for blood pressure (BP) or who had BP levels that were extremely high or low (ie, SBP levels <70 mm Hg or >270 mm Hg and DBP levels <30 mm Hg or >150 mm Hg) (eFigure in the Supplement). After exclusions, the final sample comprised 898 929 young and middle-aged adults. Participants with missing data on covariates (including geographic region of residence, educational level, employment status, occupation, marital status, household income, current smoking status, and current alcohol use) were analyzed as a separate subgroup that was categorized as unknown.

### Data Collection and Variables

Blood pressure was measured twice (after 5 minutes of quiet rest in a seated position at an interval of 1 minute) on each participant’s upper right arm using an electronic BP monitor (Omron HEM-7430; Omron Corp); measurement was performed by trained staff according to a standard operating procedure (eMethods in the Supplement). Participants were advised to stop smoking 15 minutes before the BP measurement and to turn off their mobile phones during the BP measurement. Both of the BP values and their means were recorded. If the difference between the two SBP measurements was greater than 10 mm Hg, a third BP measurement was performed; in such cases, the mean SBP and DBP were calculated using the last 2 measurements. The mean SBP and DBP values were used for all analyses.

Information on the receipt of antihypertensive, hypoglycemic, hypolipidemic, and antiplatelet medications within the past 2 weeks was collected during an in-person interview. Data regarding the participants’ sociodemographic characteristics, health behaviors, medical histories, and cardiovascular risk factors were also recorded during these in-person interviews. Height and weight were measured according to standard protocols, and body mass index was calculated as weight in kilograms divided by height in meters squared.

Because this study was performed in a Chinese cohort, we used the Chinese Guidelines for the Management of Hypertension^22^ to define hypertension and classify different hypertension subtypes. Hypertension was defined as a self-reported previous diagnosis of hypertension or receipt of antihypertensive medication in the past 2 weeks or as a mean SBP level of 140 mm Hg or higher or a mean DBP level of 90 mm Hg or higher at the screening visit. Isolated systolic hypertension was defined as a mean SBP level of 140 mm Hg or higher and a mean DBP level of less than 90 mm Hg. Isolated diastolic hypertension (IDH) was defined as a mean SBP level of less than 140 mm Hg and a mean DBP level of 90 mm Hg or higher, and systolic-diastolic hypertension (SDH) was defined as a mean SBP level of 140 mm Hg or higher and a mean DBP level of 90 mm Hg or higher, regardless of the participant’s treatment status. Controlled hypertension was defined as a self-reported previous diagnosis of hypertension or receipt of antihypertensive medication and an SBP level of less than 140 mm Hg and a DBP level of less than 90 mm Hg. Participants who did not have a history of receiving antihypertensive medication and who had an SBP level of less than 140 mm Hg and a DBP level of less than 90 mm Hg were defined as having normotension.

Participants were considered to be aware of having hypertension if they responded yes to the question, “Have you ever been diagnosed with hypertension?” Participants were considered to have received treatment for hypertension if they reported receiving an antihypertensive medication (including western or traditional Chinese medications) currently or within the last 2 weeks. Obesity was defined as a body mass index of 28 kg/m^2^ or higher, which was in accordance with the recommendations of the Working Group on Obesity in China.^23^

### Statistical Analysis

We estimated the prevalence of ISH among the overall study participants and among those with hypertension, and we compared their characteristics with individuals who had other hypertension subtypes. We also described the distribution of SBP levels among men and women with ISH across different age groups. Next, we assessed the awareness of having hypertension by sex and SBP level among individuals with ISH who had not received treatment. We then developed multivariable generalized linear mixed models with a logit link function and township-specific random intercepts (to control for geographic autocorrelation) to identify individual characteristics that were independently associated with ISH prevalence and awareness. We compared participants with ISH with those with normotension, IDH, and SDH using separate models. Explanatory variables included participants’ age, sex, marital status, annual household income, educational level, health insurance status, geographic region of residence, current smoking status, current alcohol use, obesity, physician-diagnosed diabetes, and previous cardiovascular events (myocardial infarction or stroke).

All analyses were conducted using R software, version 3.33 (R Foundation for Statistical Computing), and SAS software, version 9.4 (SAS Institute), with *P* < .05 considered statistically significant. Data were analyzed from May to November 2019.

## Results

Among 898 929 young and middle-aged adults included in the final sample, the mean (SD) age was 43.8 (3.9) years; 548 657 participants (61.0%) were women, and 235 138 participants (26.2%) had hypertension (Table 1). A total of 62 819 participants (26.7% of those with hypertension, or 7.0% of the total sample) had ISH, and 172 319 participants (73.3% of those with hypertension, or 19.2% of the total sample) had other types of hypertension (Table 2). Among those with other types of hypertension, 35 448 individuals (20.6%, or 3.9% of the total sample) had IDH, 116 682 individuals (67.7%, or 13.0% of the total sample) had SDH, and 20 189 individuals (11.7%, or 2.2% of the total sample) had controlled hypertension. Based on age and sex standardization of our results compared with data from all of the 31 provinces included in the 2010 Chinese census, the prevalence of overall hypertension and ISH among young and middle-aged adults was 24.0% and 7.0%, respectively.

**Table 1.** Characteristics of 898 929 Young and Middle-Aged Adults With and Without Isolated Systolic Hypertension

Characteristic	Participants, No. (%)				
	Normotension	Hypertension			
		ISH	IDH	SDH	Controlled
Total participants	663 791 (73.8)	62 819 (7.0)	35 448 (3.9)	116 682 (13.0)	20 189 (2.3)
Age range, y					
35-39	129 083 (19.5)	5455 (8.7)	5465 (15.4)	12 228 (10.5)	1663 (8.2)
40-44	234 368 (35.3)	17 705 (28.2)	12 035 (34.0)	34 876 (29.9)	5291 (26.2)
45-49	300 340 (45.3)	39 659 (63.1)	17 948 (50.6)	69 578 (59.6)	13 235 (65.6)
Sex					
Male	240 027 (36.2)	21 402 (34.1)	21 355 (60.2)	58 558 (50.2)	8930 (44.2)
Female	423 764 (63.9)	41 417 (65.9)	14 093 (39.8)	58 124 (49.8)	11 259 (55.8)
Urbanity of residence					
Urban	278 786 (42.0)	22 471 (35.8)	13 062 (36.8)	43 221 (37.0)	8153 (40.4)
Rural	383 932 (57.8)	40 229 (64.0)	22 310 (62.9)	73 258 (62.8)	11 995 (59.4)
Unknown	1073 (0.2)	119 (0.2)	76 (0.2)	203 (0.2)	41 (0.2)
Geographic region of residence					
Eastern	229 657 (34.6)	26 249 (41.8)	11 774 (33.2)	42 887 (36.8)	8227 (40.7)
Central	177 127 (26.7)	16 544 (26.3)	9613 (27.1)	32 362 (27.7)	5488 (27.2)
Western	256 823 (38.7)	20 022 (31.9)	14 059 (39.7)	41 408 (35.5)	6473 (32.1)
Unknown	184 (0.03)	4 (0.006)	2 (0.006)	25 (0.02)	1 (0.006)
Educational level					
≤Primary school	186 939 (28.2)	21 978 (35.0)	9686 (27.3)	35 024 (30.0)	5685 (28.2)
Middle school	244 910 (36.9)	24 860 (39.6)	13 494 (38.1)	44 633 (38.3)	7679 (38.0)
High school	119 659 (18.0)	9222 (14.7)	6203 (17.5)	19 636 (16.8)	3377 (16.7)
≥College	102 738 (15.5)	5907 (9.4)	5640 (15.9)	15 617 (13.4)	3294 (16.3)
Unknown	9545 (1.4)	852 (1.4)	425 (1.2)	1772 (1.5)	154 (0.8)
Employment status					
Employed	591 174 (89.1)	55 624 (88.5)	32 031 (90.4)	103 809 (89.0)	17 349 (85.9)
Unemployed	11 393 (1.7)	1072 (1.7)	753 (2.1)	2603 (2.2)	573 (2.8)
Retired	8702 (1.3)	1002 (1.6)	345 (1.0)	1532 (1.3)	442 (2.2)
Homemaker	39 293 (5.9)	4073 (6.5)	1667 (4.7)	6299 (5.4)	1455 (7.2)
Unknown	13 229 (2.0)	1048 (1.7)	652 (1.8)	2439 (2.1)	370 (1.8)
Occupation					
Farming	276 252 (41.6)	31 355 (49.9)	15 377 (43.4)	52 730 (45.2)	7885 (39.1)
Nonfarming	374 310 (56.4)	30 416 (48.4)	19 419 (54.8)	61 513 (52.7)	11 934 (59.1)
Unknown	13 229 (2.0)	1048 (1.7)	652 (1.8)	2439 (2.1)	370 (1.8)
Annual household income, yuan^a^					
<10 000	95 939 (14.5)	9785 (15.6)	5273 (14.9)	17 758 (15.2)	2518 (12.5)
10 000-50 000	362 481 (54.6)	35 645 (56.7)	19 404 (54.7)	64 746 (55.5)	10 670 (52.9)
>50 000	142 499 (21.5)	11 413 (18.2)	7729 (21.8)	23 527 (20.2)	5150 (25.5)
Unknown	62 872 (9.5)	5976 (9.5)	3042 (8.6)	10 651 (9.1)	1851 (9.2)
Marital status					
Married	637 608 (96.1)	60 357 (96.1)	33 930 (95.7)	111 512 (95.6)	19 308 (95.6)
Widowed, separated, divorced, or single	18 460 (2.8)	1816 (2.9)	1165 (3.3)	3683 (3.2)	707 (3.5)
Unknown	7723 (1.2)	646 (1.0)	353 (1.0)	1487 (1.3)	174 (0.9)
Health insurance status					
Insured	646 926 (97.5)	61 592 (98.0)	34 586 (97.6)	113 772 (97.5)	19 788 (98.0)
Uninsured	6096 (0.9)	495 (0.8)	312 (0.9)	1008 (0.9)	157 (0.8)
Unknown	10 978 (1.7)	744 (1.2)	561 (1.6)	1938 (1.7)	244 (1.2)
Medical history					
Myocardial infarction	1344 (0.2)	234 (0.4)	144 (0.4)	625 (0.5)	338 (1.7)
Stroke	1993 (0.3)	607 (1.0)	378 (1.1)	1906 (1.6)	679 (3.4)
Diabetes	47 632 (7.2)	8498 (13.5)	5024 (14.2)	19 638 (16.8)	4068 (20.1)
Cardiovascular risk factors					
Current smoking	118 126 (17.8)	10 742 (17.1)	11 029 (31.1)	30 759 (26.4)	4876 (24.2)
Current alcohol use	97 917 (14.8)	10 355 (16.5)	10 834 (30.6)	32 105 (27.5)	4297 (21.3)
Obesity	70 161 (10.6)	13 213 (21.0)	9617 (27.1)	36 293 (31.1)	5771 (28.6)

Abbreviations: IDH, isolated diastolic hypertension; ISH, isolated systolic hypertension; SDH, systolic-diastolic hypertension.

^a^ The average conversion rate in 2019 was 6.91 yuan to $1.00.

**Table 2.** Prevalence of Isolated Systolic Hypertension Among Young and Middle-Aged Adults With Hypertension by Individual Characteristics

Characteristic	All participants with hypertension, No.	Participants with ISH		Participants without ISH	
		No./total No. (%)	95% CI	No./total No. (%)	95% CI
Total participants	235 138	62 819/235 138 (26.7)	26.5-26.9	172 319/235 138 (73.3)	73.1-73.5
Age range, y					
35-39	24 811	5455/24 811 (22.0)	21.5-22.5	19 356/24 811 (78.0)	77.5-78.5
40-44	69 907	17 705/69 907 (25.3)	25.0-25.7	52 202/69 907 (74.7)	74.4-75.0
45-49	140 420	39 659/140 420 (28.2)	28.0-28.5	100 761/140 420 (71.8)	71.5-72.0
Sex					
Male	110 245	21 402/110 245 (19.4)	19.2-19.7	88 843/110 245 (80.6)	80.4-80.8
Female	124 893	41 417/124 893 (33.2)	32.9-33.4	83 476/124 893 (66.8)	66.6-67.1
Urbanity of residence					
Urban	86 907	22 471/86 907 (25.9)	25.6-26.2	64 436/86 907 (74.1)	73.9-74.4
Rural	147 792	40 229/147 792 (27.2)	27.0-27.5	107 563/147 792 (72.8)	72.6-73.0
Unknown	439	119/439 (27.1)	23.0-31.3	320/439 (72.9)	68.7-77.1
Geographic region of residence					
Eastern	89 137	26 249/89 137 (29.4)	29.2-29.8	62 888/89 137 (70.6)	70.3-70.9
Central	64 007	16 544/64 007 (25.8)	25.5-26.2	47 463/64 007 (74.2)	73.8-74.5
Western	81 962	20 022/81 962 (24.4)	24.1-24.7	61 940/81 962 (75.6)	75.3-75.9
Unknown	32	4/32 (12.5)	1.0-24.0	28/32 (87.5)	76.0-99.0
Educational level					
≤Primary school	72 373	21 978/72 373 (30.4)	30.0-30.7	50 395/72 373 (69.6)	69.3-70.0
Middle school	90 666	24 860/90 666 (27.4)	27.1-27.7	65 806/90 666 (72.6)	72.3-72.9
High school	38 438	9222/38 438 (24.0)	23.6-24.4	29 216/38 438 (76.0)	75.6-76.4
≥College	30 458	5907/30 458 (19.4)	19.0-19.8	24 551/30 458 (80.6)	80.2-81.1
Unknown	3203	852/3203 (26.6)	25.1-28.1	2351/3203 (73.4)	71.9-74.9
Employment status					
Employed	208 813	55 624/208 813 (26.6)	26.5-26.8	153 189/208 813 (73.4)	73.2-73.6
Unemployed	5001	1072/5001 (21.4)	20.3-22.6	3929/5001 (78.6)	77.4-79.7
Retired	3321	1002/3321 (30.2)	28.6-31.7	2319/3321 (69.8)	68.3-71.4
Homemaker	13 494	4073/13 494 (30.2)	29.4-31.0	9421/13 494 (69.8)	69.0-70.6
Unknown	4509	1048/4509 (23.2)	22.0-24.5	3461/4509 (76.8)	75.5-78.0
Occupation					
Farming	107 347	31 355/107 347 (29.2)	28.9-29.5	75 992/107 347 (70.8)	70.5-71.1
Nonfarming	123 282	30 416/123 282 (24.7)	24.4-24.9	92 866/123 282 (75.3)	75.1-75.6
Unknown	4509	1048/4509 (23.2)	22.0-24.5	3461/4509 (76.8)	75.5-78.0
Annual household income, yuan^a^					
<10 000	35 334	9785/35 334 (27.7)	27.2-28.2	25 549/35 334 (72.3)	71.8-72.8
10 000-50 000	130 465	35 645/130 465 (27.3)	27.1-27.6	94 820/130 465 (72.7)	72.4-72.9
>50 000	47 819	11 413/47 819 (23.9)	23.5-24.3	36 406/47 819 (76.1)	75.8-76.5
Unknown	21 520	5976/21 520 (27.8)	27.2-28.4	15 544/21 520 (72.2)	71.6-72.8
Marital status					
Married	225 107	60 357/225 107 (26.8)	26.6-27.0	164 750/225 107 (73.2)	73.0-73.4
Widowed, separated, divorced, or single	7371	1816/7371 (24.6)	23.7-25.6	5555/7371 (75.4)	74.4-76.4
Unknown	2660	646/2660 (24.3)	22.7-25.9	2014/2660 (75.7)	74.1-77.3
Health insurance status					
Insured	229 738	61 592/229 738 (26.8)	26.6-27.0	168 146/229 738 (73.2)	73.0-73.4
Uninsured	1913	483/1913 (25.3)	23.3-27.2	1430/1913 (74.8)	72.8-76.7
Unknown	3487	744/3487 (21.3)	20.0-22.7	2743/3487 (78.7)	77.3-80.0
Medical history					
Myocardial infarction	1341	234/1341 (17.4)	15.4-19.5	1107/1341 (82.6)	80.5-84.6
Stroke	3570	607/3570 (17.0)	15.8-18.2	2963/3570 (83.0)	81.8-84.2
Diabetes	37 228	8498/37 228 (22.8)	22.4-23.3	28 730/37 228 (77.2)	76.8-77.6
Cardiovascular risk factors					
Current smoking	57 406	10 742/57 406 (18.7)	18.4-19.0	46 664/57 406 (81.3)	81.0-81.6
Current alcohol use	57 591	10 355/57 591 (18.0)	17.7-18.3	47 236/57 591 (82.0)	81.7-82.3
Obesity	64 894	13 213/64 894 (20.4)	20.1-20.7	51 681/64 894 (79.6)	79.3-80.0

Abbreviation: ISH, isolated systolic hypertension.

^a^ The average conversion rate in 2019 was 6.91 yuan to $1.00.

### Prevalence and Characteristics

Among 235 138 young and middle-aged participants with hypertension, 62 819 individuals (26.7%) had ISH (mean [SD] age, 45.0 [3.5] years; 41 417 women [65.9%]). A total of 54 463 individuals (86.7%) with ISH had not received treatment. Overall, the prevalence of ISH was higher among older age groups (39 659 of 140 420 individuals [28.2%; 95% CI, 28.0%-28.5%] aged 45-49 years vs 5455 of 24 811 individuals [22.0%; 95% CI, 21.5%-22.5%] aged 35-39 years), women (41 417 of 124 893 women [33.2%; 95% CI, 32.9%-33.4%] vs 21 402 of 110 245 men [19.4%; 95% CI, 19.2%-19.7%]), participants residing in the eastern region of China (26 249 of 89 137 individuals [29.4%; 95% CI, 29.2%-29.8%] in the eastern region vs 20 022 of 81 962 individuals [24.4%; 95% CI, 24.1%-24.7%] in the western region), participants with lower educational levels (21 978 of 72 373 individuals [30.4%; 95% CI, 30.0%-30.7%] with a primary school education or lower vs 5907 of 30 458 individuals [19.4%; 95% CI, 19.0%-19.8%] with a college education or higher), participants who were employed (55 624 of 208 813 employed individuals [26.6%; 95% CI, 26.5%-26.8%] vs 1072 of 5001 unemployed individuals [21.4%; 95% CI, 20.3%-22.6%]), and participants who were farmers (31 355 of 107 347 farmers [29.2%; 95% CI, 28.9%-29.5%] vs 30 416 of 123 282 nonfarmers [24.7%; 95% CI, 24.4%-24.9%]) (Table 2). In addition, approximately 1 in 5 participants with obesity (13 213 of 64 894 individuals [20.4%; 95% CI, 20.1%-20.7%]), diabetes (8498 of 37 228 individuals [22.8%; 95% CI, 22.4%-23.3%]), current smoking (10 742 of 57 406 individuals [18.7%; 95% CI, 18.4%-19.0%]), current alcohol use (10 355 of 57 591 individuals [18.0%; 95% CI, 17.7%-18.3%]), previous myocardial infarction (234 of 1341 individuals [17.4%; 95% CI, 15.4%-19.5%]), and previous stroke (607 of 3570 individuals [17.0%; 95% CI, 15.8%-18.2%]) had ISH.

**Figure 1.**
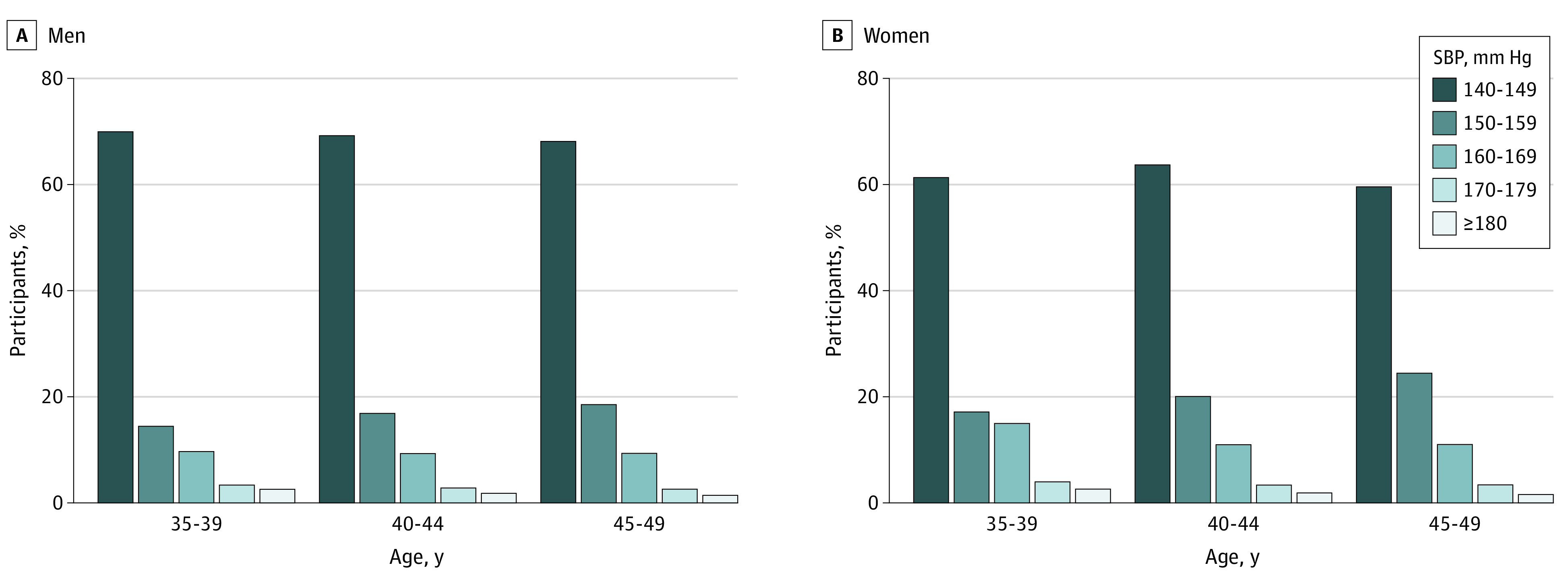
Distribution of Systolic Blood Pressure (SBP) Among Participants With Isolated Systolic Hypertension Across Age Groups

Overall, approximately 9737 of 62 819 individuals (15.5%; 95% CI, 15.3%-15.8%) with ISH (both treated and untreated) had an SBP level of 160 mm Hg or higher. The proportion of participants with ISH who had an SBP level of 160 mm Hg or higher was greater among women than among men across all ages (for ages 35-39 years, 630 of 2923 women [21.6%; 95% CI, 20.1%-23.1%] vs 395 of 2532 men [15.7%; 95% CI, 14.2%-17.1%]; for ages 40-44 years, 1716 of 10 855 women [16.3%; 95% CI, 15.5%-16.9%] vs 953 of 6850 men [13.9%; 95% CI, 13.1%-14.8%]; for ages 45-49 years, 4417 of 27 639 women [16.0%; 95% CI, 15.6%-16.4%] vs 1606 of 12 020 men [13.3%; 95% CI, 12.8%-14.0%]) (Figure 1).

Compared with individuals with normotension (n = 663 791), participants with ISH were more likely to be obese (70 161 individuals [10.6%; 95% CI, 10.5%-10.6%] vs 13 213 individuals [21.0%; 95% CI, 20.7%-21.4%], respectively), currently use alcohol (97 917 [14.8%; 95% CI, 14.7%-14.8%] vs 10 355 individuals [16.5%; 95% CI, 16.2%-16.8%]), have diabetes (47 632 individuals [7.2%; 95% CI, 7.1%-7.2%] vs 8498 individuals [13.5%; 95% CI, 13.3%-13.8%]), and have a previous history of stroke (1993 individuals [0.3%; 95% CI, 0.3%-0.3%] vs 607 individuals [1.0%; 95% CI, 0.9%-1.0%]) (Table 1). Compared with participants who had other hypertension subtypes, such as IDH (n = 35 448) and SDH (n = 116 682), participants with ISH were more likely to be aged 45 to 49 years (39 659 individuals [63.1%; 95% CI, 62.8%-63.5%] with ISH vs 17 948 individuals [50.6%; 95% CI, 50.1%-51.2%] with IDH and 69 578 individuals [59.6%; 95% CI, 59.3%-59.9%] with SDH), female (41 417 individuals [65.9%; 95% CI, 65.6%-66.3%] with ISH vs 14 093 individuals [39.8%; 95% CI, 39.2%-40.3%] with IDH and 58 124 individuals [49.8%; 95% CI, 49.5%-50.1%] with SDH), have an educational level of primary school or lower (21 978 individuals [35.0%; 95% CI, 34.6%-35.4%] with ISH vs 9686 individuals [27.3%; 95% CI, 26.9%-27.8%] with IDH and 35 024 individuals [30.0%; 95% CI, 29.8%-30.3%] with SDH), be farmers (31 355 individuals [49.9%; 95% CI, 49.5%-50.3%] with ISH vs 15 377 individuals [43.4%; 95% CI, 42.9%-43.9%] with IDH and 52 730 individuals [45.2%; 95% CI, 44.9%-45.5%] with SDH), and reside in eastern regions of China (26 249 individuals [41.8%; 95% CI, 41.4%-42.2%] with ISH vs 11 774 individuals [33.2%; 95% CI, 32.7%-33.7%] with IDH and 42 887 individuals [36.8%; 95% CI, 36.5%-37.0%] with SDH) (Table 1).

In our multivariable analysis, when compared with participants with normotension, participants who were female, were older, were obese, currently used alcohol, had lower annual household income and lower educational levels, did not have health insurance, had a history of diabetes or cardiovascular events, and resided in eastern or central regions had a greater likelihood of ISH; however, marital status was not a substantial factor (Table 3). When compared with participants with IDH and SDH, participants with ISH were more likely to be older, be female, and reside in central or eastern regions but were less likely to have higher household income, educational levels of college or higher, previous cardiovascular events, and obesity and to currently smoke and use alcohol.

**Table 3.** Mixed-Effects Multivariable Regression Models

Characteristic	Odds ratio (95% CI)			
	Prevalence of ISH among participants with untreated hypertension			Awareness of ISH among participants with untreated ISH
	Model 1: ISH vs normotension	Model 2: ISH vs IDH	Model 3: ISH vs SDH	
Age				
Per 5 y	1.72 (1.70-1.74)	1.39 (1.37-1.42)	1.07 (1.06-1.09)	1.22 (1.16-1.28)
Sex				
Male	1 [Reference]	1 [Reference]	1 [Reference]	1 [Reference]
Female	1.14 (1.12-1.17)	2.41 (2.33-2.49)	1.58 (1.54-1.62)	1.36 (1.24-1.50)
Marital status				
Unmarried	1 [Reference]	1 [Reference]	1 [Reference]	1 [Reference]
Married	0.99 (0.94-1.03)	1.01 (0.94-1.09)	1.04 (0.99-1.10)	0.99 (0.83-1.18)
Annual household income, yuan^a^				
<10 000	1 [Reference]	1 [Reference]	1 [Reference]	1 [Reference]
10 000-50 000	0.95 (0.93-0.98)	1.01 (0.97-1.05)	1.01 (0.98-1.04)	0.83 (0.75-0.91)
>50 000	0.85 (0.83-0.88)	0.93 (0.89-0.98)	0.96 (0.93-1.00)	0.85 (0.75-0.96)
Educational level				
<College	1 [Reference]	1 [Reference]	1 [Reference]	1 [Reference]
≥College	0.71 (0.69-0.73)	0.74 (0.71-0.78)	0.77 (0.74-0.79)	1.12 (0.99-1.27)
Health insurance status				
Uninsured	1 [Reference]	1 [Reference]	1 [Reference]	1 [Reference]
Insured	1.22 (1.11-1.34)	1.17 (1.00-1.36)	1.08 (0.97-1.20)	1.04 (0.71-1.53)
Cardiovascular risk factors				
Current smoking	0.95 (0.92-0.97)	0.95 (0.92-0.99)	0.95 (0.92-0.98)	1.27 (1.14-1.43)
Current alcohol use	1.21 (1.18-1.25)	0.75 (0.73-0.78)	0.71 (0.69-0.73)	1.31 (1.19-1.45)
Diabetes	1.79 (1.74-1.84)	1.05 (1.01-1.10)	0.87 (0.85-0.90)	1.58 (1.44-1.72)
Obesity	2.10 (2.06-2.14)	0.75 (0.73-0.78)	0.62 (0.60-0.63)	1.49 (1.38-1.61)
Previous cardiovascular event(s)	2.23 (2.07-2.41)	0.99 (0.89-1.10)	0.68 (0.63-0.74)	3.92 (3.19-4.81)
Geographic region of residence				
Western	1 [Reference]	1 [Reference]	1 [Reference]	1 [Reference]
Central	1.19 (1.17-1.22)	1.18 (1.14-1.22)	1.06 (1.03-1.09)	1.06 (0.97-1.15)
Eastern	1.54 (1.51-1.57)	1.61 (1.56-1.67)	1.30 (1.27-1.33)	0.81 (0.75-0.88)

Abbreviations: IDH, isolated diastolic hypertension; ISH, isolated systolic hypertension; SDH, systolic-diastolic hypertension.

^a^ The average conversion rate in 2019 was 6.91 yuan to $1.00.

### Awareness

**Figure 2.**
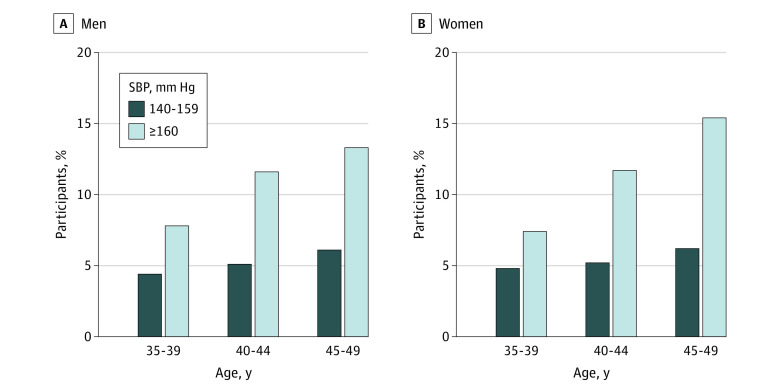
Awareness of the Presence of Hypertension Among Participants With Untreated Isolated Systolic Hypertension by Age and Systolic Blood Pressure Level SBP indicates systolic blood pressure.

Among the 54 463 participants with ISH who had not received treatment (86.7% of the total participants with ISH), only 3682 individuals (6.8%) were aware of having hypertension, whereas 1736 of the 30 365 participants (5.7%) with IDH who had not received treatment and 15 526 of the 87 586 participants (17.7%) with SDH who had not received treatment were aware of having hypertension (eTable in the Supplement). Among participants with ISH who had not received treatment, awareness rates were higher among older age groups (2458 individuals [7.3%; 95% CI, 7.1%-7.6%] aged 45-49 years and 967 individuals [6.1%; 95% CI, 5.7%-6.4%] aged 40-44 years vs 257 individuals [5.1%; 95% CI, 4.5%-5.8%] aged 35-39 years), women (2474 women [7.0%; 95% CI, 6.7%-7.2%] vs 1208 men [6.4%; 95% CI, 6.0%-6.7%]), and individuals who lived in rural areas (2453 individuals [7.0%; 95% CI, 6.8%-7.3%] in rural areas vs 1221 individuals [6.3%; 95% CI, 5.9%-6.6%] in urban areas) and central or western regions (1089 individuals [7.7%; 95% CI, 7.3%-8.2%] in central regions and 1287 individuals [7.3%; 95% CI, 6.9%-7.7%] in western regions vs 1306 individuals [5.8%; 95% CI, 5.5%-6.1%] in eastern regions). Approximately 10% or less of participants with ISH who had 1 or more cardiovascular risk factor, including current smoking (706 individuals [7.5%; 95% CI, 7.0%-8.1%]), current alcohol use (715 individuals [7.9%; 95% CI, 7.3%-8.5%]), obesity (958 individuals [9.1%; 95% CI, 8.6%-9.7%]), and diabetes (679 individuals [10.3%; 95% CI, 9.6%-11.1%]), were aware of having hypertension. In addition, approximately 25% or less of participants with ISH who had a previous history of myocardial infarction (32 individuals [22.5%; 95% CI, 16.0%-30.3%]) or stroke (88 individuals [25.4%; 95% CI, 20.9%-30.3%]) were aware of having hypertension (eTable in the Supplement). Although awareness rates increased with age, they remained low among both sexes even after stratification by SBP level (Figure 2). For example, among individuals with an SBP level of 160 mm Hg or higher, awareness rates were 7.8% for men and 7.4% for women aged 35 to 39 years, and awareness rates were 13.3% for men and 15.4% for women aged 45 to 49 years.

In our multivariable analysis, older age, female sex, and the presence of cardiovascular risk factors (such as current smoking and alcohol use, obesity, history of diabetes, and previous cardiovascular events) remained significant factors associated with the awareness of having hypertension (Table 3). However, marital status, educational level, health insurance status, and geographic region were not substantial factors.

## Discussion

In this large population-based cross-sectional study, we found that ISH was present in approximately 1 of 4 young and middle-aged adults (26.7%) with hypertension in China, most of whom (86.7%) had not received treatment; only 6.8% of those who had not received treatment were aware of having hypertension. In addition, approximately 1 in 6 individuals (15.5%) with untreated ISH had an SBP level of 160 mm Hg or higher; however, awareness rates remained low (≤15.4%) in this group. Moreover, even among individuals with 1 or more cardiovascular risk factors and a history of cardiovascular events, approximately 90% and 75% of individuals with ISH, respectively, remained unaware of having hypertension.

Our study expands the existing literature on ISH in several ways. First, to our knowledge, our study is one of the largest to describe the current prevalence and characteristics of young and middle-aged adults with ISH in China, which allowed us to explore associations across a variety of diverse subgroups. We found that ISH was present in 26.7% of young and middle-aged adults with hypertension in China, which is consistent with the previously reported prevalence among cohorts from non-Chinese populations.^2,3,9,24^ Young and middle-aged adults with ISH in China were more likely to be older, female, and obese and to currently use alcohol, have diabetes, and have a history of previous cardiovascular events compared with those with normotension, which is consistent with the associations previously reported in the literature for non-Chinese populations, particularly from studies in the US and Europe.^3,8,9,24,25^ In addition, we found that young and middle-aged adults with ISH were more likely to have lower socioeconomic status and reside in the central or eastern regions of China than individuals with normotension, which, to our knowledge, has not been previously reported. These factors may be associated with the participants’ health care access, motivation to make healthy lifestyle choices, adherence to preventive health guidelines, and management of comorbidities associated with hypertension.^26^

Second, our study is the first, to our knowledge, to describe the awareness of having hypertension among young and middle-aged adults with ISH in a contemporary Chinese population. We found that only 6.8% of untreated individuals with ISH were aware of having hypertension, and awareness rates remained low even among those with high SBP levels (≤15.4% among adults with SBP≥160 mm Hg) or a history of previous cardiovascular events (≤25.4%). These awareness rates are substantially lower than those for the overall population of individuals with hypertension in China (44.7%).^27^ Younger adults are more difficult to reach through traditional clinic-based preventive programs because they may be less aware of the long-term benefits of early control of cardiovascular risk factors and therefore less likely to be in contact with the health system and less motivated to make lifestyle changes.^28,29,30^ In addition, given that the clinical importance of the treatment of ISH in younger adults has been questioned in the past^31,32^ and that most previous studies of ISH have focused on older individuals, there are currently no recommendations for the management of ISH in younger adults.^22,24,33^ Thus our findings may be a reflection of the lack of clinical data in this population, and they highlight the need for clinical trials among this population.

### Limitations

This study has several limitations. First, patients who received treatment for ISH and decreased their SBP level to less than 140 mm Hg were classified as having controlled hypertension, which could have underestimated the burden of ISH in China. However, very few individuals who originally had ISH would have been classified as having controlled hypertension given the low hypertension treatment rates, and the even lower control rates, in China. Second, some individuals with hypertension could have experienced a preferential improvement in their DBP levels and may have been included in the ISH group, leading to overestimation of the rates for ISH. However, overestimation is unlikely to have been a substantial factor, as most individuals (86.7%) in the ISH group had not received treatment. Third, our current study design did not permit us to examine ISH in adults younger than 35 years. Fourth, because the China PEACE MPP is a large-scale population-based screening project, BP was only measured at a single visit. Considering the effect of regression to the mean, we may have overestimated the prevalence of hypertension and ISH. However, the effect of regression to the mean should not be substantial. Fifth, we used a convenience sample rather than a nationally representative sample for large-scale recruitment, which may have limited the generalizability of our findings to China despite their consistency with the age- and sex-standardized prevalence of ISH in the 2010 Chinese census data. Additionally, inclusion of this sample could have resulted in overestimation of the awareness and treatment rates because these participants would have been more likely to have contact with the health system.

## Conclusions

In this large population-based cross-sectional study, we found that ISH was present in approximately 30% of young and middle-aged adults with hypertension in China, most of whom remained unaware of having hypertension. These results highlight the increasing need for improved awareness of ISH in this population and the need for better evidence-based guidance for the management of ISH among younger individuals.

## Notes

Supplement. **eMethods.** Standard Operating Procedure for Blood Pressure Measurement in the China PEACE Million Persons Project **eTable. **Awareness of Having Hypertension Among Participants With Untreated Isolated Systolic Hypertension Compared With Participants With Untreated Systolic Diastolic Hypertension and Participants With Untreated Isolated Diastolic Hypertension, by Population Characteristics **eFigure.** Flowchart of Study Participant Selection **eReferences**
