# Supplementary material for: Assessment of Prevalence, Awareness, and Characteristics of Isolated Systolic Hypertension Among Younger and Middle-Aged Adults in China
Source: JAMA Netw Open. 2020 Dec 8;3(12):e209743. doi: 10.1001/jamanetworkopen.2020.9743 (PMC7724558; doi:10.1001/jamanetworkopen.2020.9743)
Supplement: Supplement. — eMethods. Standard Operating Procedure for Blood Pressure Measurement in the China PEACE Million Persons Project eTable. Awareness of Having Hypertension Among Participants With Untreated Isolated Systolic Hypertension Compared With Participants With Untreated Systolic Diastolic Hypertension and Participants With Untreated Isolated Diastolic Hypertension, by Population Characteristics eFigure. Flowchart of Study Participant Selection eReferences [file jamanetwopen-e209743-s001.pdf]

Supplementary Online Content

Mahajan S, Feng F, Hu S, et al. Assessment of prevalence, awareness, and characteristics of isolated systolic hypertension among younger and middle-aged adults in China. *JAMA Netw Open*. 2020;3(12):e209743. doi:10.1001/jamanetworkopen.2020.9743

eMethods. Standard Operating Procedure for Blood Pressure Measurement in the China PEACE Million Persons Project

eTable. Awareness of Having Hypertension Among Participants With Untreated Isolated Systolic Hypertension Compared With Participants With Untreated Systolic Diastolic Hypertension and Participants With Untreated Isolated Diastolic Hypertension, by Population Characteristics

eFigure. Flowchart of Study Participant Selection

eReferences

This supplementary material has been provided by the authors to give readers additional information about their work.

eMethods. Standard Operating Procedure for Blood Pressure Measurement in the China PEACE Million Persons Project

Blood pressure (BP) was measured twice for every participant. Both BP values as well as their mean were recorded. If the difference between the two SBP measurements was >10 mmHg, a third BP measurement was done, and the mean SBP and DBP were calculated using the last two measurements in such cases. The mean SBP and DBP were used for all analyses. BP was measured after 5 minutes of quiet rest in the seated position, at an interval of 1 minute, on participants' right upper arm, using an electronic BP monitor (Omron HEM-7430; Omron Corporation, Kyoto, Japan) by trained staff according to a standard operation procedure. Participants were advised to stop smoking 15 minutes before the BP measurement, avoid any obvious position changes and emotional excitement, and if possible, empty their bladder. In the process of BP measurement, participants were asked to relax their mind and avoid exerting force, speaking, and moving limbs. When measuring BP, participants were requested to turn off their mobile phones to avoid the interference of electromagnetic field that may affect the accuracy of measurement results. If there was serious arrhythmia or the arm was too thin (without children's cuff), the ordinary mercury sphygmomanometer was to be used for BP measurement.

We provided a cuff (17–36 cm) for each blood pressure monitor. Although arm circumference was not measured, a larger cuff (32–45 cm) was used for those with large arm circumference. In the standard operating procedure for BP measurement, we described that if BP can't be measured because of large arm circumference (such as obesity), a larger cuff (32-45 cm) should be used, and if arm circumference is too small to be measured, calibrated mercurial sphygmomanometer should be used.

The BP monitor was calibrated when manufactured, as stated in the product manual. In addition, all devices were regularly maintained, including cleaning the device, checking battery

power, and replacing the battery when necessary. All devices were calibrated once a year to ensure consistency of measurements. Calibration was performed by the local sites or local bureaus of quality and technical monitoring, according to the Verification Regulation of Non-invasive Automated Sphygmomanometers (JJG 692-2010). This regulation was issued by the General Administration of Quality Supervision, Inspection and Quarantine of China. The major calibration indicators include range and error of static pressure measurement. The BP monitor was considered to meet the requirement if it can measure from 0 to 260 mm Hg and the measurement errors should be within ±4 mm Hg compared with a standard manometer.

The accuracy of this BP monitor has been validated in different populations, including adolescents and older adults according to the international protocol of the European Society of Hypertension.[1-4] In addition, this monitor meets the standards of the Association for the Advancement of Medical Instrumentation (AAMI),[5] an international organization that aims to enhance the development, use, and maintenance of healthcare technology and medical devices.

eTable. Awareness of Having Hypertension Among Participants With Untreated Isolated Systolic Hypertension Compared With Participants With Untreated Systolic Diastolic Hypertension and Participants With Untreated Isolated Diastolic Hypertension, by Population Characteristics

Characteristics, n (%, 95% CI)	Awareness among untreated ISH participants, N=54473	Awareness among untreated SDH participants, N=87586	Awareness among untreated IDH participants, N=30365
**No. (%)**	3682 (6.8)	15 526 (17.7)	1736 (5.7)
**Age (years)**			
35-39	257 (5.1% [4.5-5.8])	1624 (16.2% [15.4-16.9])	271 (5.4% [4.8-6.1])
40-44	967 (6.1% [5.7-6.4])	4601 (16.8% [16.3-17.2])	555 (5.3% [4.8-5.7])
45-49	2458 (7.3% [7.1-7.6])	9301 (18.6% [18.2-18.9])	910 (6.1% [5.8-6.5])
**Sex**			
Men	1208 (6.4% [6.0-6.7])	8054 (18.0% [17.7-18.4])	1125 (6.2% [5.8-6.5])
Women	2474 (7.0% [6.7-7.2])	7472 (17.4% [17.1-17.8])	611 (5.0% [4.6-5.4])
**Urbanity**			
Urban	1221 (6.3% [5.9-6.6])	5430 (16.6% [16.2-17.0])	652 (5.9% [5.4-6.3])
Rural	2453 (7.0% [6.8-7.3])	10,067 (18.4% [18.1-18.7])	1080 (5.6% [5.3-6.0])
Unknown	8 (8.2% [3.6-15.6])	29 (19.7% [13.6-27.1])	4 (6.3% [1.8-15.5])
**Region**			
Eastern	1306 (5.8% [5.5-6.1])	4851 (15.2% [14.8-15.6])	505 (5.2% [4.7-5.6])
Central	1089 (7.7% [7.3-8.2])	4596 (19.4% [18.9-19.9])	525 (6.5% [6.0-7.1])
Western	1287 (7.3% [6.9-7.7])	6076 (19.1% [18.6-19.5])	706 (5.6% [5.2-6.1])
**Education**			
Primary school or low	1497 (7.9% [7.5-8.3])	5035 (19.3% [18.8-19.8])	453 (5.3% [4.8-5.8])
Middle school	1307 (6.1% [5.8-6.4])	5520 (16.5% [16.1-16.9])	614 (5.4% [5.0-5.8])
High School	474 (5.9% [5.4-6.4])	2495 (16.7% [16.1-17.3])	274 (5.2% [4.6-5.8])
College or above	350 (6.9% [6.2-7.6])	2230 (19.0% [18.3-19.7])	372 (7.9% [7.2-8.7])
Unknown	54 (7.1% [5.4-9.1])	246 (17.3% [15.3-19.3])	23 (6.0% [3.8-8.9])
**Employment**			
Employed	3208 (6.6% [6.4-6.9])	13,791 (17.6% [17.3-17.9])	1571 (5.7% [5.4-6.0])
Unemployed	73 (8.3% [6.6-10.3])	346 (19.6% [17.8-21.6])	38 (6.5% [4.6-8.8])
Retired	53 (6.5% [4.9-8.5])	183 (17.1% [14.9-19.5])	16 (6.0% [3.5-9.6])
Housework	273 (8.0% [7.1-9.0])	836 (18.6% [17.5-19.8])	76 (5.5% [4.4-6.9])
Unknown	75 (8.0% [6.3-9.9])	370 (19.9% [18.1-21.8])	35 (6.1% [4.3-8.4])
**Occupation**			
Farmer	1949 (7.1% [6.8-7.5])	7337 (18.6% [18.3-19.0])	701 (5.2% [4.8-5.6])
Non-farmer	1658 (6.3% [6.0-6.6])	7819 (16.9% [16.5-17.2])	1000 (6.2% [5.8-6.5])
Unknown	75 (8.0% [6.3-9.9])	370 (19.9% [18.1-21.8])	35 (6.1% [4.3-8.4])
**Household income (Yuan/year)**			
<10,000	678 (7.9% [7.3-8.5])	2733 (20.0% [19.4-20.7])	251 (5.3% [4.7-6.0])

Characteristics, n (%, 95% CI)	Awareness among untreated ISH participants, N=54473	Awareness among untreated SDH participants, N=87586	Awareness among untreated IDH participants, N=30365
10,000-50,000	1962 (6.3% [6.1-6.6])	8157 (16.9% [16.6-17.2])	899 (5.4% [5.1-5.7])
>50,000	604 (6.2% [5.8-6.7])	3077 (17.5% [17.0-18.1])	423 (6.6% [6.0-7.3])
Unknown	438 (8.4% [7.6-9.1])	1559 (19.2% [18.3-20.0])	163 (6.2% [5.3-7.2])
**Marital status**			
Married	3531 (6.7% [6.5-7.0])	14,844 (17.7% [17.5-18.0])	1673 (5.8% [5.5-6.0])
Widowed, separated, divorced, single	110 (7.1% [5.9-8.5])	500 (18.3% [16.9-19.8])	47 (4.9% [3.6-6.4])
Unknown	41 (7.1% [5.2-9.6])	182 (15.4% [13.4-17.6])	16 (5.1% [2.9-8.1])
**Health insurance status**			
Insured	3606 (6.8% [6.5-7.0])	15,166 (17.8% [17.5-18.0])	1697 (5.7% [5.5-6.0])
Uninsured	27 (6.3% [4.2-9.0])	120 (15.6.4% [13.1-18.4])	15 (5.9% [3.3-9.5])
Unknown	49 (7.3% [5.5-9.6])	240 (15.7% [13.9-17.7])	24 (4.9% [3.2-7.3])
**Medical history**			
Myocardial infarction	32 (22.5% [16.0-30.3])	115 (38.2% [32.7-44.0])	23 (25.0% [16.6-35.1])
Stroke	88 (25.4% [20.9-30.3])	371 (40.2% [37.0-43.4])	49 (23.0% [17.5-29.2])
Diabetes mellitus	679 (10.3% [9.6-11.1])	2859 (21.6% [20.9-22.3])	298 (7.5% [6.7-8.4])
**Cardiovascular risk factors**			
Current smoker	706 (7.5% [7.0-8.1])	4482 (19.4% [18.9-19.9])	576 (6.2% [5.7-6.7])
Current drinker	715 (7.9% [7.3-8.5])	4982 (20.4% [19.9-20.9])	613 (6.7% [6.2-7.2])
Obesity	958 (9.1% [8.6-9.7])	5147 (20.5% [20.0-21.0])	527 (6.8% [6.3-7.4])

Abbreviations: CI, confidence interval; IDH, isolated diastolic hypertension; ISH, isolated systolic hypertension; SDH, systolic diastolic hypertension

eFigure. Flowchart of Study Participant Selection

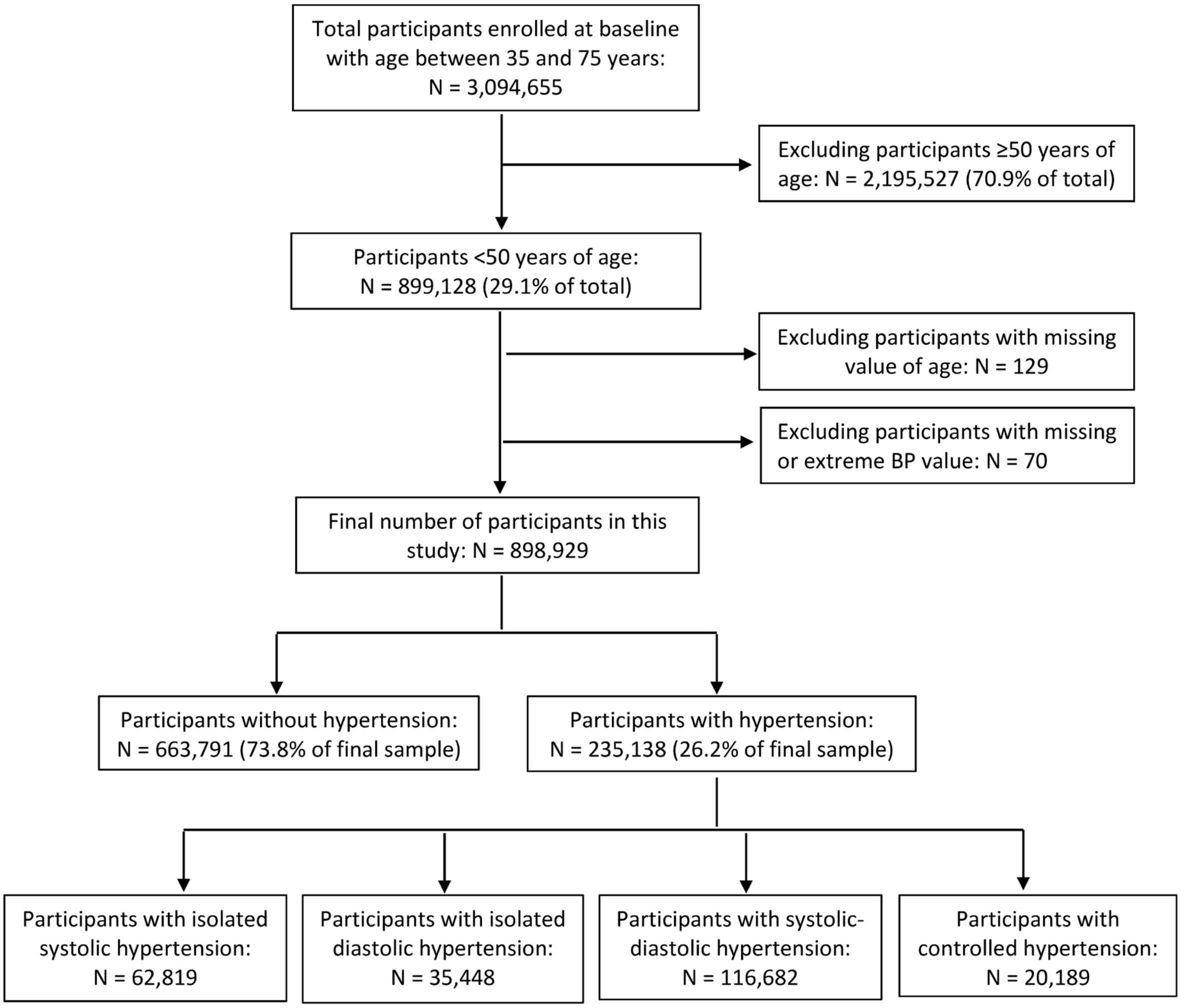

© 2020 Mahajan S et al. *JAMA Network Open*.

eReferences

1. Takahashi H, Yoshika M and Yokoi T. Validation of three automatic devices for the self-measurement of blood pressure according to the European Society of Hypertension International Protocol revision 2010: the Omron HEM-7130, HEM-7320F, and HEM-7500F. Blood pressure monitoring. 2015;20:92-7.

2. Takahashi H, Yoshika M and Yokoi T. Validation of two automatic devices for the self-measurement of blood pressure according to the ANSI/AAMI/ISO81060-2:2009 guidelines: the Omron BP765 (HEM-7311-ZSA) and the Omron BP760N (HEM-7320-Z). Vascular health and risk management. 2015;11:49-53.

3. Omboni S, Riva I, Giglio A, Caldara G, Groppelli A and Parati G. Validation of the Omron M5-I, R5-I and HEM-907 automated blood pressure monitors in elderly individuals according to the International Protocol of the European Society of Hypertension. Blood pressure monitoring. 2007;12:233-42.

4. Christofaro DG, Fernandes RA, Gerage AM, Alves MJ, Polito MD and Oliveira AR. Validation of the Omron HEM 742 blood pressure monitoring device in adolescents. Arquivos brasileiros de cardiologia. 2009;92:10-5.

5. Lewelling J. AAMI Standards: A History of Ensuring Medical Technology Quality and Performance. Biomedical instrumentation & technology. 2017;51:210-211.
